# A screening questionnaire for convulsive seizures: A three-stage field-validation in rural Bolivia

**DOI:** 10.1371/journal.pone.0173945

**Published:** 2017-03-16

**Authors:** Loretta Giuliano, Calogero Edoardo Cicero, Elizabeth Blanca Crespo Gómez, Sandra Padilla, Elisa Bruno, Mario Camargo, Benoit Marin, Vito Sofia, Pierre-Marie Preux, Marianne Strohmeyer, Alessandro Bartoloni, Alessandra Nicoletti

**Affiliations:** 1 Department of Medical and Surgical Sciences and Advanced Technologies “G.F. Ingrassia”, Section of Neurosciences, University of Catania, Catania, Italy; 2 Hospital Universitario Hernández Vera, Santa Cruz, Bolivia; 3 Center of Anthropological Researches of the Teko Guaraní, Gutierrez, Bolivia; 4 Department of Neurology, Guy’s & St Thomas’ NHS Foundation Trust, London, United Kingdom; 5 The Bolivian League Against Epilepsy, Santa Cruz, Bolivia; 6 INSERM, U1094, Tropical Neuroepidemiology, Limoges, France; 7 Department of Experimental and Clinical Medicine, Infectious and Tropical Diseases Unit, University of Florence, Florence, Italy; University of Rome Tor Vergata, ITALY

## Abstract

**Introduction:**

Epilepsy is one of the most common neurological diseases in Latin American Countries (LAC) and epilepsy associated with convulsive seizures is the most frequent type. Therefore, the detection of convulsive seizures is a priority, but a validated Spanish-language screening tool to detect convulsive seizures is not available. We performed a field validation to evaluate the accuracy of a Spanish-language questionnaire to detect convulsive seizures in rural Bolivia using a three-stage design. The questionnaire was also administered face-to-face, using a two-stage design, to evaluate the difference in accuracy.

**Methods:**

The study was carried out in the rural communities of the Gran Chaco region. The questionnaire consists of a single screening question directed toward the householders and a confirmatory section administered face-to-face to the index case. Positive subjects underwent a neurological examination to detect false positive and true positive subjects. To estimate the proportion of false negative, a random sample of about 20% of the screened negative underwent a neurological evaluation.

**Results:**

792 householders have been interviewed representing a population of 3,562 subjects (52.2% men; mean age 24.5 ± 19.7 years). We found a sensitivity of 76.3% (95% CI 59.8–88.6) with a specificity of 99.6% (95% CI 99.4–99.8). The two-stage design showed only a slightly higher sensitivity respect to the three-stage design.

**Conclusion:**

Our screening tool shows a good accuracy and can be easily used by trained health workers to quickly screen the population of the rural communities of LAC through the householders using a three-stage design.

## Introduction

Epilepsy is one of the most prevalent non communicable neurologic diseases, with an estimated aggregate burden of around 0.7% of the total global disease burden [1]. It affects approximately 70 million people worldwide [2] of whom ¾ live in low and middle-income countries (LMIC) where the treatment gap (TG) ranges from over 50% to 75% with higher levels found in rural areas [3]. The higher prevalence and incidence rates of epilepsy in LMIC reflect differences in several risk factors [4–9].

During the last decades, several epidemiological surveys have been carried out in LMIC to estimate the prevalence of epilepsy, often using a two-stage design. In two-stage neuroepidemiological studies, during the screening phase, the population is interviewed face-to-face through the use of validated screening questionnaires and then evaluated by the specialists [10]. However, this method is costly to implement in large populations since the first stage takes considerable time and the second stage requires qualified medical personnel who often have to assess a large number of false positive subjects.

Several screening instruments have been developed and validated, rarely in the field and more often in a hospital context, to detect epilepsy in rural setting [11,12].

As recently highlighted by the WHO, the detection of epilepsy associated with convulsive seizures (EACS) is a priority in rural areas of LMIC, since it is associated with higher comorbidity, injury and mortality than non-convulsive epilepsy [13,14]. Moreover, EACS can be easily identified by community health workers (CHW) because of their clear clinical manifestations and consequently treated with relatively available drugs [15]. Furthermore, minor seizures such as partial seizures are more difficult to detect at community-level [11,16]. Nevertheless, to the best of our knowledge, only two screening instruments have been developed in order to specifically detect convulsive epilepsy, showing a sensitivity between 48.6 and 72.1% with very high specificity level [17,18]. Both questionnaires have been developed to be used in a three-stage design: in the first stage one or two preliminary questions are directed toward a senior member of each household by a non-medical fieldworker; in a second confirmatory phase, only people with a history of convulsions identified by the householders are interviewed face-to-face by trained CHW, using a more detailed questionnaire; in the third stage only the positive subjects at the second confirmatory phase undergo a clinical evaluation in order to confirm the diagnosis. Such a design allows a reduction of cost and time related to the face-to-face interview of the entire population.

It has been estimated that about 5 million people with epilepsy (PWE) live in Latin American Countries (LAC) [9], representing a considerable and often untreated health problem. In this region the estimated median lifetime epilepsy (LTE) prevalence is 15.8/1,000 (10.7/1,000 for active epilepsy) with a median TG of 60.6% and 77.8% in the rural area [4]. In rural Bolivia EACS is the most frequent type (about 60%) [16] and it is associated with the highest stigma [19,20]. In the Gran Chaco area, the life-time prevalence of EACS is 7.2/1,000 (6.6/1,000 for active EACS) with a crude incidence risk of 55.4/100,000 and a TG for active EACS of 70.2% [21]. To the best of our knowledge, validated Spanish-language questionnaires for convulsive epilepsy are not available in the literature. It is known that the validity of a screening tool is strictly dependent on the setting of use, so a questionnaire should be validated in the setting where it will be used. We performed a field validation in the rural communities of the Gran Chaco region in Bolivia of a Spanish-language screening questionnaire [18] to detect convulsive seizures using a three-stage design. The screening questionnaire is a slightly modified version of the Anand’s tool. We have also evaluated the different accuracy of the screening instrument when used in a two-stage or three-stage study design.

## Methods

### Study area and population

Bolivia is a low-middle-income country with high levels of poverty especially in rural areas, where 40% of the population lives. The southeast region of Bolivia is part of the “Gran Chaco”, a subtropical area also including Argentina and Paraguay. The ethnic group living in the study area is mainly represented by native Guaraní people, living in poor dwellings located in rural communities often reachable only by rural roads, without running water or electricity, and with a local economy based on agriculture and animal husbandry. Most of the population speaks both Spanish and Guaraní. The study was carried out in two Departments of the Gran Chaco region: the areas of Lagunillas and Gutierrez, located in the Cordillera Province, Department of Santa Cruz de la Sierra and the areas of Huacaya and Machareti, located in the Luis Calvo province, in the Department of Chuquisaca. A random sample of rural communities from these two different areas of the Gran Chaco region was selected using a cluster sampling method, considering each community as a cluster, in order to achieve the required sample size. We used as sampling list the “Censo de Poblacion y Vivienda 2012” [22].

### Sample size calculation

The sample size was estimated according to the following formula, taking into account the expected sensitivity of the questionnaire:

$${\text{n}}_{\text{S}}=\frac{{\text{Z}}_{\frac{\alpha }{2}}^{2}\hat{\text{S}}\;(1-\hat{\text{S}})}{{\text{d}}^{2}\text{x Prev}}$$

Considering α = 0.05, Z α2 is inserted by 1.96; S and Prev are the pre-determined values of sensitivity and prevalence of disease respectively and d^2^ is the precision of the estimate [23,24]. According to this formula, a sample size of 2,921 subjects was needed to reach 90% sensitivity [11] with a precision of 13% and considering an EACS prevalence of 7.2/1,000 [21]. Furthermore, on the basis of previous population-based studies in the same area [16,19,21], a response rate of 80% was expected, so in order to add a 20% of expected non- responders, 584 subjects were added to the sample, reaching a total sample size of 3,505 subjects.

### Screening tool

We used a modified Spanish version of the screening instrument developed by Anand et al. [18]. The questionnaire was translated and modified with the help of a local Guaraní anthropologist (SP) and the comprehension of the single items was pre-tested during a pilot study carried out in two rural communities. The screening questionnaire consists of two sections (S1 Table): the first section (stage I) is a single question administered to the householders (or the most reliable person available in the family) enquiring about the presence of convulsive seizures in one or more members of the family. The second section (stage II) is administered face-to-face to the positive subjects identified during stage I and is composed by the same screening question administered to the householder plus six more specific confirmatory questions that explore symptoms related to tonic-clonic seizures. For subjects under 12 years of age, or not available at the time of the screening phase, both stage I and II were indirectly performed using a proxy responder.

Subjects who were found to be positive at at least one stage II question underwent a complete neurological evaluation (stage III) for the confirmation of the diagnosis.

The questionnaires were administered by 18 Guaraní nursing students attending the last year of their course at the Tekove Katu School, over the December 2015 and June 2016 period. They had previously been trained for three days by three neurologists (one local neurologist EBCG, and two Italian neurologists AN, CEC) at Tekove Katu School in the community of Gutierrez in October 2015. The training focused on the clinical manifestations of convulsive seizures with a comprehensive description and different video examples. They were also trained to administer the questionnaire in two different sessions: at first, they administered the questionnaires to each other and discussed their doubts with the neurologists and the anthropologist. After that, they administered the questionnaire in a rural community under the supervision of the neurologists and the anthropologist.

### Validation phase

All positive subjects at the screening phase (stage I and II) underwent a neurological evaluation in order to detect true positive (TP) and false positive (FP) subjects. The neurological examination (stage III) was performed in the field by three neurologists (one local neurologist EBCG and two Italian neurologists LG, CEC). A member of the *Asamblea del Pueblo Guaraní* (APG), who could speak Guaraní, assisted in the field work. Convulsive seizures were defined according to the 1993 ILAE definition. EACS was defined according to worldwide accepted WHO definition as “a chronic brain disorder of various etiologies characterized by spontaneous attacks of discontinuous contractions of the body musculature due to excessive discharge of cerebral neurons” [25,26]. We used the 1981 classification of seizures [27]. In order to estimate the proportion of false negative (FN) and true negative (TN) subjects, the neurologists evaluated a random sample of about 20% of negative subjects, from a sample of randomly selected communities. The neurologists were blinded for the previous results of the screening. The validation was performed in June 2016.

Furthermore, in order to compare the accuracy of the classic two-stage study design, in which the questionnaire is administered face-to-face to the entire study population (except for subjects less than 12 years of age), with the accuracy of the three-stage study design, the screening question was also administered face-to-face to all the adult subjects available at the time of the screening. Thus, in this sample of subjects we evaluated the accuracy of both the two-stage and the three-stage study design. The informed consent was obtained by the nursing students during the screening phase, after a complete explanation of the study. Written consent was obtained, whenever possible, using a Spanish language informed consent. In case of illiteracy oral consent was obtained and a written list was used to record the subjects giving the consent. The study was approved by the Bolivian Society of Neurology and by the ethics committee of the University Hospital “Policlinico Vittorio Emanuele” of Catania, in Italy (41/2016/PO), which also approved the consent procedure. The study was developed in accordance with the STARD guidelines (S1 Checklist) [28].

### Statistical analysis

The questionnaires were collected, coded, anonymized and entered in an ad-hoc created database (Epidata 2.0.5.17) by a trained local worker. Data cleaning was also performed before the data analysis considering both range and consistency checks. Qualitative variables were described as percentages and quantitative variables as mean ± standard deviation (SD). Sensitivity, specificity, positive and negative predictive values (PPV and NPV) were calculated considering the three-stage design. In a sub-group of subjects we have also estimated the accuracy of the two-stage design. The prevalence of convulsive seizures and EACS was calculated. Point prevalence was estimated considering the 1^st^ January 2016 as prevalence day. 95% confidence intervals (CI) were estimated. Data were analyzed using STATA 12 software packages (version 12.0, College Station, TX).

## Results

### Validation

At the end of the study 792 householders had been gone through the stage I interview, representing a population of 3,562 subjects (1,860 men [52.2%]; mean age 24.5 ± 19.7 years), of whom 1,118 (31.4%) were under 12 years of age. The proxy responders were represented by mothers or wives for 2,185 cases (61.3%), fathers or husbands for 1,148 (32.2%), and others such as grandparents, brothers or sisters for the remaining 229 subjects (6.5%). Overall 42 subjects (1.2%) out of the 45 positive subjects at stage I, screened positive at stage II, of whom 29 were classified as TP and 13 as FP after the neurological evaluation, giving a prevalence for convulsive seizures of 10.7/1,000 (95% CI 7.6–14.6). Among the TP subjects, 20 fulfilled the diagnostic criteria for EACS giving a prevalence of 8.1/1,000, (95% CI 5.5–11.7), while six were classified as febrile convulsions and three had had a single seizure.

**Fig 1:**
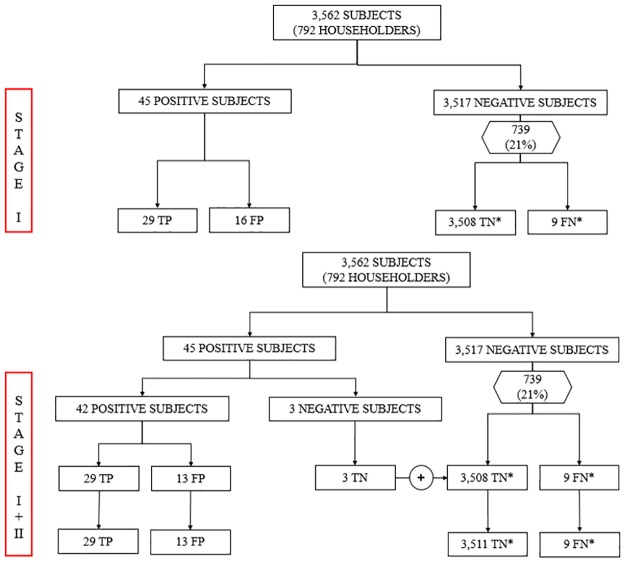
Flow chart describing the validation process. TP, true positive; FP, false positive; TN, true negative; FN, false negative. * expected values.

Baseline characteristics of the study population are shown in Table 1. The validation process is shown in Fig 1.

**Table 1:** Baseline characteristics of the subjects screened positive and negative after the two-stage design.

	Screened positive		Screened negative		Total number	
	N = 42		N = 3,520		N = 3,562	
	N	%	N	%	N	%
**Sex (M)**	25	59.5%	1,835	52.1%	1,860	52.2
**Age** (mean ± SD)	29.7 ± 20.8		24.4 ± 19.7		24.5 ± 19.7	
**Education**						
*None*	7	16.7	627	17.8	634	17.8
*Primary*	20	47.6	1,679	47.7	1,699	47.7
*Secondary*	12	28.6	1,049	29.8	1,061	29.8
*Other*	3	7.1	165	4.7	168	4.7
**Occupation**						
*Farmer*	10	23.8	588	16.7	598	16.8
*Housewife*	6	14.3	765	21.7	771	21.6
*Teacher*	1	2.4	30	0.9	31	0.9
*Health personnel*	0	0	12	0.3	12	0.3
*Other*	25	59.5	2,125	60.4	2,150	60.4
**Area**						
*Gutierrez*	8	19.0	408	11.6	416	11.7
*Huacaya*	22	52.4	1,247	35.4	1,269	35.6
*Lagunillas*	7	16.7	1,278	36.3	1,285	36.1
*Machareti*	5	11.9	587	16.7	592	16.6

N, number; SD, standard deviation.

In order to estimate the proportion of FN, of the 3,520 subjects (98.8%) screened negative at the questionnaire, 739 (21%) subjects were randomly selected and underwent a neurological evaluation. Out of the 739 subjects two (0.3%; 95% CI 0–0.6) were classified as FN. In particular, one was a 14-year-old boy suffering from generalized epilepsy with monthly tonic-clonic seizures, started one year before while the second FN subject was a 15-year-old girl presenting a partial epilepsy with secondary generalization with the last generalized tonic-clonic seizure occurring more than five years before.

According to our sample proportion of FN (0.3%), in the whole sample (3,520 negative subjects) nine subjects are expected to be FN, giving a sensitivity for the detection of convulsive seizures of 76.3% (95% CI 59.8–88.6) and a specificity of 99.6% (95% CI 99.4–99.8) (Table 2).

**Table 2:** Values of accuracy of the questionnaire.

*Best scenario*^a^	**Sensitivity % (95% CI)**	**Specificity % (95% CI)**	**PPV % (95% CI)**	**NPV % (95% CI)**	**TP**	**FP**	**TN**^c^	**FN**^c^
Three-stage design	76.3 (59.8–88.6)	99.6 (99.4–99.8)	69.0 (52.9–82.4)	99.7 (99.5–99.9)	29	13	3511	9
*Worst scenario*^b^	**Sensitivity % (95% CI)**	**Specificity % (95% CI)**	**PPV % (95% CI)**	**NPV % (95% CI)**	**TP**	**FP**	**TN**^c^	**FN**^c^
Three-stage design	58.0 (43.2–71.8)	99.6 (99.4–99.8)	69.0 (52.9–82.4)	99.4 (99.1–99.6)	29	13	3499	21

CI, confidence intervals; PPV, positive predictive value; NPV, negative predictive value; TP, true positive; FP, false positive; TN, true negative; FN, false negative.

^a^ False negative (n = 9) have been estimated on the basis of the sample proportion (0.3%).

^b^ False negative (n = 21) have been estimated on the basis of the upper limit of the 95% CI of the sample proportion (0.6%).

^c^ expected values.

However, considering the maximum number of expected FN subjects (n = 21), according to the upper level of the 95% CI (0.6%), the *worst scenario*, the complete questionnaire achieved a sensitivity of 58.0% (95% CI 43.2–71.8) with a specificity of 99.6% (95% CI 99.4–99.8), as shown in Table 2.

The accuracy of each single confirmatory question of stage II are shown in Table 3.

**Table 3:** Performances of the six confirmatory questions of the questionnaire.

	Sensitivity % (95% CI)	Specificity % (95% CI)	PPV % (95% CI)	NPV % (95% CI)
**Question 1**	50.0 (29.1–70.9)	66.7 (29.9–92.5)	80.0 (51.9–95.7)	33.3 (13.3–59.0)
**Question 2**	58.3 (36.6–77.9)	90.9 (58.7–99.8)	99.3 (68.0–99.8)	50.0 (27.2–72.8)
**Question 3**	76.0 (54.9–90.6)	58.3 (27.7–84.8)	79.2 (57.8–92.9)	53.8 (5.1–80.8)
**Question 4**	50.0 (29.9–70.1)	88.9 (51.7–99.7)	92.9 (66.1–99.8)	38.1 (18.1–61.6)
**Question 5**	82.6 (61.2–95.0)	10.0 (0.2–44.5)	67.9 (47.6–84.1)	20.0 (0.51–71.6)
**Question 6**	76.9 (56.3–91.0)	25.0 (5.5–57.2)	69.0 (49.2–84.7)	33.3 (7.5–70.1)

CI, confidence interval; PPV, positive predictive value; NPV, negative predictive value.

### Accuracy of the two-stage versus three-stage study design

The screening question was administered face-to-face to 1,434 adult subjects of the study population because 982 were not available at the time of the screening. Considering the two-stage method and including in this analysis an overall population of 2,552 subjects, consisting of 1,434 adults who were directly interviewed and 1,118 subjects under 12 who could be only indirectly interviewed, we obtained a sensitivity of 72.4% (95% CI 52.8–87.3), a specificity of 99.6% (95% CI 99.3–99.8) a PPV of 70.0% (95% CI 50.6–85.3) and a NPV of 99.7% (95% CI 99.4–99.9). In the same population of 2,552 subjects, when considering the three-stage design, sensitivity was 68.0% (95% CI 46.5–85.0) specificity 99.8% (95% CI 99.5–99.9), PPV 73.9% (95% CI 51.6–89.8) and NPV 99.7% (95% CI 99.4–99.9).

## Discussion

During the last decades several screening questionnaires have been developed to detect PWE in the general population, often using a two stage survey, in order to determine the prevalence and incidence of epilepsy [11,29]. In two-stage surveys the population is screened with a questionnaire, often administered by trained CHW during the first phase and the diagnosis is confirmed by a neurological evaluation in the second phase [10]. Therefore, the choice of the screening tool is a critical point in any epidemiological study, representing a potential pitfall. Even if a perfect level of sensitivity and specificity is often difficult to achieve, and at any rate is not required for a screening tool, the knowledge of the sensitivity and specificity is important to quantify the potential inaccuracy of a screening test. For this reason, it is recommended to use validated and standardized screening instruments and the validation of a screening questionnaire is a critical point. Therefore, the translation of a screening tool is a crucial process that should always be undertaken with great care and ideally a translated questionnaire should be re-validated in the setting in which it will be used.

A recent systematic review of screening questionnaires for epilepsy used in population-based cohort studies has highlighted a high variability confirming the need for further high-quality validation studies [29].

The detection of convulsive seizures is a priority in rural areas of LMIC. For this reason the WHO has recently wondered if “*convulsive epilepsy can be diagnosed at first level care by a non-specialist health care provider in LMIC settings*” [11]. This question arises from the awareness that EACS constitutes a greater health problem than non-convulsive epilepsy since it is associated with greater stigma and mortality [13,19,21]. Another important issue that arises in detecting epilepsy in LMIC is *“Who Would Be Questioned*?*”* [30]. Usually in two-stage neuroepidemiological studies, during the screening phase, a face-to-face interview is performed, with high logistic and time costs. A suitable alternative can be represented by the administration of the questionnaire to the householders using a three-stage design.

Up to date only two questionnaires have been developed and validated to detect convulsive seizures (Table 4) [17,18].

**Table 4:** Characteristics of the existing questionnaires for convulsive seizures.

Study	Country	Language	Target condition	Validation	Accuracy
Ngugi et al. 2012	Kenya	Kigiriama	Active epilepsy	Field validation in a rural setting	Sensitivity: 48.6% (95% CI 31.4–66.0); Specificity: 100%
Anand et al. 2005	India	Indian	Generalized tonic-clonic seizures	Mixed field and hospital-based validation	Sensitivity: 72.1% (95% CI 65.2–78.1); Specificity: 100% (95% CI 84–100)

Both tools, developed for a three-stage design, consist of one or two generic screening questions directed to the householder and a second confirmatory section directed to the index case. Such design offers several advantages reducing the cost and timing related to the face-to-face interview, but also allowing to reduce the number of false positive subjects, thus increasing the specificity level at the second stage and reducing the number of subjects to be visited by the neurologists, rarely available in these areas.

However, only the screening tool developed by Ngugi has been field validated in a rural area reporting a quite low sensitivity of 48.6% with a specificity of 100% and an estimated cost reduction of about 40% in respect to the two-stage design. According to the authors, the stigma-related non-response could have been the main cause of the low sensitivity of the three-stage design [17]. On the other hand, the questionnaire developed by Anand showed a good sensitivity level of 72.1% with a specificity of 100% [18]. However, it was mainly validated in a hospital setting and only a sample of patients and controls were enrolled at community level. The generalization of estimates obtained by hospital-based validation is limited by selection bias due to the overrepresentation of cases with more severe forms of epilepsy and with a greater awareness of the condition. Furthermore, in a hospital-based context, only subjects who have been diagnosed will be enrolled, while people who lack knowledge of epilepsy may fail to seek treatment and therefore are not captured in hospital-based validations. Thus, these studies provide less accurate estimates than population-based validations [10,17]. For these reasons in rural settings of LMIC the field validation method is probably the best way to perform a validation in order to develop a useful instrument, as much as adapted as possible to the reality in which it will be used.

Although it has been estimated that about 5 million PWE live in LAC, no validated Spanish-language questionnaire to detect convulsive seizures exists. We validated a Spanish-language questionnaire to detect convulsive seizures in the rural population. Our instrument showed a good level of sensitivity (76.3%) and specificity (99.6%) with values close to those obtained by Anand. The higher level of sensitivity respect to the Ngugi questionnaire (48.6%) could be probably due to the greater awareness among the Guaraní population of convulsive seizures known as “*manu-manu”* in Guaraní language [16,19–21,31]. However we cannot exclude a lower level of perceived stigma in our population [17].

Another important consideration arising from our study is related to the comparison of the accuracy between the classic two-stage study design and the three-stage design. To the best of our knowledge this is the first study in which such comparison has been made. Only one study has previously assessed the concordance between the screening question when directed toward the householder or directly administered, but considered different neurological disorders [30]. According to our findings, sensitivity achieved by the face-to-face interview in the two-stage design was only slightly higher compared to the level achieved by the three-stage design (72.4% *versus* 68.0%), while the level of specificity, PPV and NPV was almost the same. From our point of view, the about 4 percentage points gained in the two-stage design do not justify the difference in terms of cost and time.

Furthermore, in the two-stage design people who are not present at the time of the screening interview cannot be included in the study. In our study, in fact, we were able to perform a face-to-face interview only for 1,434 adults because 982 were not available at the time of the screening. Moreover, it should be underlined that in rural communities most men are farmer who often work in the fields far from their house and can be away for a couple of months due to seasonal works such as during the sugarcane harvesting time. However, we are aware that this analysis is affected by the smaller size of the sample investigated that has led to less precise estimates with a wider range of 95% CI.

Another important limit of our study is represented by the detection of false negative subjects that has been performed just in a sample of about 20% of the screened negative subjects, an approach already used by Placencia in Ecuador [10]. The neurological evaluation of the entire screened population certainly represents the gold standard and would have been the most suitable option, in order to perform a more reliable and complete validation. However, in this rural setting neurologists are rarely available and the communities are spread out over a large area often very far from one another, so the clinical evaluation of the entire screened population was not feasible due to cost and time reasons. Nevertheless, taking into account the upper limit of the 95% CI, also considering the *worst scenario*, our sensitivity level is not lower than 58.0% keeping a very high specificity level (99.6%).

Concerning the FN subjects, it is important to underline that they were missed both at stage I and II, the direct and indirect interview respectively. One possible explanation could be in one case the absence of tonic-clonic seizures for more than 5 years (thus considered non-active EACS), while for the other case the recent onset of seizures, starting less than one year before. It should be noted that for both cases the proxy responder at stage I was the father. As previously reported, men are often away from home and thus could not be totally aware regarding family issues. On the other hand, according to our experience in rural communities, the mothers are the most common (61.3%). and accurate proxy responders because they spend most of their time at home and are better informed regarding the health status of their family members.

In conclusion, our screening tool represents a valuable instrument, with an acceptable level of sensitivity and excellent specificity that can be easily used, specially by trained health workers, in order to quickly screen the entire population living in the rural communities of LAC through the householders. Ideally, training all the community health workers, most subjects affected by EACS living in the rural communities could be rapidly detected in order to implement an appropriate treatment strategy, as recommended by the WHO [11].

## Supporting information

S1 Checklist STARD checklist. (DOCX)

S1 Table The original instrument used in the study. (DOC)
