# Supplementary material for: A screening questionnaire for convulsive seizures: A three-stage field-validation in rural Bolivia
Source: PLoS One. 2017 Mar 16;12(3):e0173945. doi: 10.1371/journal.pone.0173945 (PMC5354446; doi:10.1371/journal.pone.0173945)
Supplement: S1 Table — (DOC) [file pone.0173945.s002.doc]

**S1 Figure:** **The original instrument used in the study.**

| **Section 1** |  |
| --- | --- |
| **Screening question directed toward the householder**  *Spanish*  Alguien de su familia ha tenido ataques con perdida del conocimiento sin darse cuenta de lo que pasaba con movimientos incontrolables o involuntarios (de los brazos o de las piernas, etc.) de un minuto o màs?  *English*  Have anyone in your family had seizures with loss of consciousness without realizing what was happening, with uncontrollable or involuntary movements (arms or legs, etc.) lasting for a minute or more? | **Answer**  Si/No/No sabe  Yes/No/ I don’t know |
| **Section 2** |  |
| **Screening question directed toward the index case**  *Spanish*  Usted ha tenido ataques o le han dicho que ha tenido ataques con pérdida del conocimiento sin darse cuenta de lo que pasaba con movimientos incontrolables o involuntarios (de los brazos o de las piernas) de un minuto o màs?  *English*  Have you ever had attacks or been told you had attacks with loss of consciousness without realizing what was happening, with uncontrollable or involuntary movements (arms or legs) lasting for a minute or more?  **Confirmatory questions directed to the index case**  *Spanish*  1) Se orinó o se hizo caca durante el ataque?  2) Se mordió la lengua o se lastimó durante el ataque?  3) Se baveó durante el ataque?  4) Ha tenido ataque cuando estaba dormiendo (noche o madrugada)?  5) Se acuerda que le pasó durante el ataque?  6) Cuantas veces ha tenido este tipo de ataque?  *English*  1) Have you ever peed or pooped during the attack?  2) Have you ever bitten your tongue or got injured during the attack?  3) Have you ever drooled during the attack?  4) Have you ever had the attack while you were sleeping (night or early morning)?  5) Do you remember what happened during the attack?  6) How often did you have this kind of attack? | **Answer**  Si/No/No sabe  Yes/No/ I don’t know  **Answer**  Si/No/No sabe  Si/No/No sabe  Si/No/No sabe  Si/No/No sabe  Si/No/No sabe  Una vez/Màs que una vez/No sabe  Yes/No/ I don’t know  Yes/No/ I don’t know  Yes/No/ I don’t know  Yes/No/ I don’t know  Yes/No/ I don’t know  Once/More than once/I don’t know |
